# Exercise Training Intensity and the Fitness-Fatness Index in Adults with Metabolic Syndrome: A Randomized Trial

**DOI:** 10.1186/s40798-021-00395-7

**Published:** 2021-12-24

**Authors:** Joyce S. Ramos, Lance C. Dalleck, Mackenzie Fennell, Alex Martini, Talita Welmans, Rebecca Stennett, Shelley E. Keating, Robert G. Fassett, Jeff S. Coombes

**Affiliations:** 1 Caring Futures Institute & SHAPE Research Centre, Exercise Science and Clinical Exercise Physiology, College of Nursing and Health Sciences, Flinders University, Room 268, Sturt South, Sturt Building, Bedford Park, Adelaide, SA 5042 Australia; 2 Centre for Research on Exercise, Physical Activity and Health, School of Human Movement and Nutrition Sciences, The University of Queensland, Brisbane, QLD Australia; 3 Recreation, Exercise, and Sport Science Department, Western State Colorado University, Gunnison, CO USA

**Keywords:** Fitness fatness index, Interval training, Metabolic syndrome

## Abstract

**Background:**

Cardiorespiratory fitness and fatness (notably central obesity) are mediating factors of the metabolic syndrome (MetS) and consequent cardiovascular disease (CVD)/mortality risk. The fitness-fatness index (FFI) combines these factors and has been reported to be a better indicator of CVD and all-cause mortality risk, beyond the capacity of either fitness or fatness alone.

**Objective:**

This study sought to investigate the effects of different exercise intensities on FFI in adults with MetS.

**Methods:**

This was a sub-study of the ‘Exercise in the prevention of Metabolic Syndrome’ (EX-MET) multicentre trial. Ninety-nine adults diagnosed with MetS according to the International Diabetes Federation criteria were randomized to one of the following 16-week exercise interventions: i) moderate-intensity continuous training (MICT) at 60–70% HRpeak for 30 min/session (*n* = 34, 150 min/week); ii) 4 × 4 min bouts of high-intensity interval training at 85–95% HRpeak, interspersed with 3-min active recovery at 50–70% HRpeak (*n* = 34, 38 min/session, 114 min/week); and iii) 1 × 4 min bout of HIIT at 85–95% HRpeak (*n* = 31, 17 min/session, 51 min/week). Cardiorespiratory fitness (peak oxygen uptake, V̇O_2_peak) was determined via indirect calorimetry during maximal exercise testing and fatness was the ratio of waist circumference-to-height (WtHR). FFI was calculated as V̇O_2_peak in metabolic equivalents (METs) divided by WtHR. A clinically meaningful response to the exercise intervention was taken as a 1 FFI unit increase.

**Results:**

Seventy-seven participants completed pre and post testing to determine FFI. While there was no significant between group difference (*p* = 0.30), there was a small group x time interaction effect on FFI [*F*(2, 73) = 1.226; *η*^2^ = 0.01], with numerically greater improvements following HIIT (4HIIT, + 16%; 1HIIT, + 11%) relative to MICT (+ 7%). There was a greater proportion of participants who had a clinically meaningful change in FFI following high-volume HIIT (60%, 15/25) and low-volume HIIT (65%, 17/26) compared to MICT (38%, 10/26), but with no significant between-group difference (*p* = 0.12). A similar trend was found when a sub-analysis comparing the FFI between those with type 2 diabetes (MICT, 33%, 3/9; high-volume HIIT, 64%, 7/11; and low-volume HIIT, 58%, 7/12) and without type 2 diabetes (MICT, 41%, 7/17; high-volume HIIT, 57%, 8/14; low-volume HIIT, 71%, 10/14).

**Conclusion:**

Although there were no statistically significant differences detected between groups, this study suggests that the response to changes in FFI in adults with MetS may be affected by exercise intensity, when numerical differences between exercise groups are considered. Further research is warranted.

*Trial registration number and date of registration*: ClinicalTrials.gov NCT01676870; 31/08/2012.

## Key Points

- Low- or high-volume high-intensity interval training (HIIT) may induce a higher proportion of likely responders to a clinically significant improvement in fitness-fatness index (FFI) compared to moderate-intensity continuous training (MICT).
- A similar trend was found in a sub-analysis comparing the numerical FFI change between individuals with or without type 2 diabetes.
- The main finding of this study was that although there was no statistically significant difference between training groups detected, it is plausible that higher exercise intensity may augment responsiveness of individuals with MetS to improvements in FFI, when numerical differences between training groups are considered. Further research is warranted.

## Introduction

Metabolic syndrome (MetS) is the clustering of cardiovascular disease risk factors [1], increasing an individual’s susceptibility to type 2 diabetes (T2D) and subsequent cardiovascular disease (CVD) [2] and mortality [3]. Cardiorespiratory fitness [4] and fatness [5] are mediating factors of MetS and thus have been considered viable targets in the prevention of T2D and CVD-related mortality in those diagnosed with the syndrome. Recently, Sloan et al. [6] developed an index that combines the interaction between fitness and fatness; the fitness fatness index (FFI), calculated as cardiorespiratory fitness divided by waist circumference-to-height ratio (WtHR). This index has been reported to be a better indicator of incident T2D [6, 7], and all-cause and CVD-specific mortality risk, beyond the capacity of either fitness or fatness alone [8]. Edward and Loprinzi [8] showed that a 1-FFI-unit increase is associated with a 9% and 11% reduction in all-cause and CVD-specific mortality, respectively. FFI can therefore be considered a widely accessible clinical tool that can help practitioners better monitor the risk of developing T2D and premature mortality in those with MetS.

Interestingly, the association between an FFI increase and reduced risk of all-cause mortality has been reported to be driven more by the favourable effects of fitness [9], suggesting the importance of tailoring exercise programs towards augmenting fitness as a primary objective. The current exercise guideline of 150 min per week of moderate-intensity continuous training (MICT) has long been established as an effective intervention to improve fitness and cardiovascular risk factors constituting the MetS [10]. However, high-volume high-intensity interval training (HIIT) has been demonstrated to increase fitness more than MICT [11], specifically in people with MetS [12]. In addition, Tjonna et al. [13] have also shown that low-volume HIIT (1HIIT, 1 × 4 min interval at 90% peak heart rate [HRpeak]) improves fitness to a similar extent as high-volume HIIT (4HIIT, 4 × 4 min intervals at 90% HRpeak, interspersed by 3 min active recovery). This is an exciting finding given that time constraint is often the most cited barrier to long-term exercise adherence [14]. The impact of different exercise volumes on FFI however, has yet to be explored. The aim of this study was to therefore investigate the effects of different exercise volumes on FFI in adults with MetS. We hypothesised that low-volume HIIT will be as efficacious as high-volume HIIT and MICT in augmenting FFI in individuals with MetS. Based on our previous findings comparing people with and without T2D [15], we also aimed to determine the effect of the different training interventions on FFI in those with and without this condition.

## Methods

**Fig. 1:**
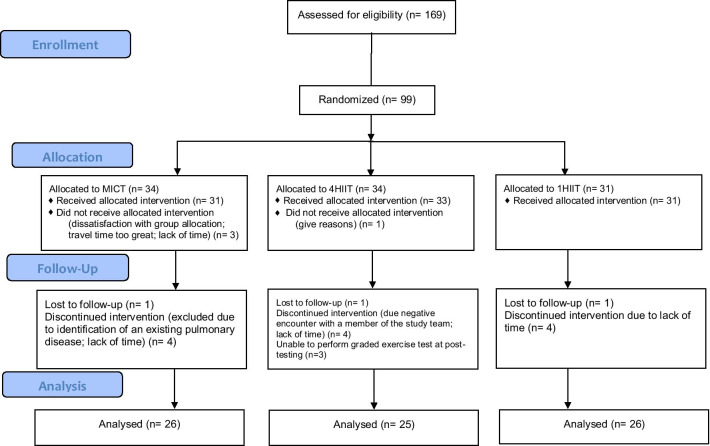
Consort Flow Diagram for FFI sub-study. 1HIIT, 1 × 4 min high-intensity interval training; 4HIIT, 4 × 4 min high- intensity interval training; MICT, moderate-intensity continuous training

Participants in this study were part of the ‘Exercise in prevention of Metabolic Syndrome (EX-MET)’ international multicentre project described previously [16]. This-sub-study investigated the change in FFI values in participants recruited from the trial site at Brisbane, Australia. Recruitment was conducted through several methods: i) a website was developed to serve as a recruitment link for social platforms and the University’s online magazine; ii) referrals from medical practitioners at the Princess Alexandra Hospital; and iii) advertising through posters, newspapers, television news and flyers disseminated across the university and local health care centres. Prospective participants were excluded if they presented with any of the following: recent myocardial infarction (last four weeks), unstable angina, uncompensated heart failure, severe valvular heart disease, uncontrolled hypertension, pulmonary disease, cardiomyopathy, and kidney failure. Written and oral consent were obtained from all participants prior to inclusion. Ninety-nine individuals diagnosed with MetS according to the International Diabetes Federation criteria [17] were included and randomized into the following exercise groups (stratified by age, sex, and centre): i) MICT (*n* = 34); ii) 4HIIT (*n* = 34); and iii) 1HIIT (*n* = 31) (Fig. 1). The randomization procedure was performed via a software employing random permuted blocks. De-identified details of participants eligible were entered into an online system to acquire group allocation.

Before and after the 16-week exercise interventions, participants underwent several tests at the university’s laboratory (Human Movement and Nutrition Sciences Building, St Lucia Campus, The University of Queensland, QLD, Australia) to assess the primary (FFI) and secondary outcome measures (MetS risk factors and body composition). Participants were instructed to refrain from strenuous activities for at least 48 h, and caffeine and alcohol for at least 24 h before each examination. All assessments were conducted at approximately the same time of the day (morning, ± 2 h). This study was approved by the Medical Research Ethics Committee, The University of Queensland (Brisbane, Australia).

### Metabolic Syndrome

To determine the participants’ eligibility for the study, the following assessments were conducted after a 12-h fast: i) brachial systolic and diastolic blood pressure; ii) fasting lipid profile and glucose-level; and iii) anthropometric measures (height, waist circumference, weight, and hip circumference). Details of these assessments have been reported previously [18].

### Fitness Fatness Index

The FFI was calculated as the ratio between cardiorespiratory fitness, expressed as the metabolic equivalent (MET), and WtHR. Waist circumference and height were measured according to the protocols presented in Coombes and Skinner [19]. Briefly, waist circumference was measured at least twice at the narrowest point between the lower costal (10th rib) border and the top of the iliac crest, perpendicular to the trunk’s long axis. The WtHR was calculated by dividing the waist circumference in cm by height in cm. Cardiorespiratory fitness depicted as the peak oxygen update (V̇O_2_peak, mL/kg/min) was assessed via indirect calorimetry using the Parvo Medics TrueOne 2400 and Metamax II system (Cortex, Leipzig, Germany) during a graded maximal exercise test. V̇O_2_peak was determined as the highest 15- second time averaged V̇O_2_, expressed relative to the participant’s mass in mL/kg/min. V̇O_2_peak in mL/kg/min was subsequently converted to METs by dividing it by 3.5 mL/kg/min. A cycle or treadmill ergometer was used during the test according to the participants’ preferred training method during the supervised exercise sessions or orthopedic limitations. In order to standardize nutrition for the test, participants were provided with a liquid nutritional supplement (Sustagen, 250 mL, Dutch Chocolate, Nestle, Gympie QLD, Australia) to consume two hours before the assessment. All tests were preceded with an 8-min warm-up which included 2 stages (stage 1 warm-up: 4 km/h at 0% incline or 50–60 revolutions per minute [rpm] at 0 W; stage 2 warm-up: 4 km/h at 4% incline or 50–60 rpm at 25 W). The speed (individualized: within 6–9 km/h) and load (2% incline or 50 W) were subsequently increased each minute until exhaustion. Standardized verbal cues were provided throughout the graded exercise test to motivate participants to reach maximal effort.

### Body Composition

Dual-energy X-ray absorptiometry (DEXA; Hologic QDR 4500 version 12.6) was used to assess pre- and post-intervention measures of body fat indices (total body and regional [android and gynoid] fat distributions [%]) and lean mass. Participants were required to be in a 12-h overnight fasted state for this assessment. Total caloric intake was monitored from baseline to post-intervention using a 3-day food diary. A diet analysis software (FoodWorks 8 Professional; Xyris Software) was subsequently used to analyse the food diary data.

### Moderate-to-Vigorous Physical Activity

Accelerometers (ActiGraph GT3X_, Pensacola, FL) were used to objectively assess average daily time spent in moderate-to vigorous physical activity (MVPA). The accelerometer was placed on the participants’ right hip during waking hours for 7 days at baseline and post-testing. The ActiLife 6.1 software was used to analyse the data in 60 s sampling frequency. Minimum wear time was determined as the accelerometer device worn: i) at least one day during a weekend day; and ii) 10 h per day for four of the 7 days. Non-wear time was determined as 60 min of consecutive zeros [20]. The time in MVPA was defined by a cut-off point of 2020 counts per minute [21].

### Training Protocol

The MICT group completed five exercise sessions per week, whilst the HIIT group trained three times per week (at least a day between sessions). All participants were required to attend two supervised sessions per week at The University of Queensland, while the remaining session/s were performed unsupervised. The unsupervised exercise sessions consisted of participant-preferred outdoor or indoor pursuits involving large muscle groups such as walking, running, or cycling. Both exercise heart rate and rating of perceived exertion (RPE) were monitored and recorded throughout the exercise sessions using a heart rate monitor (Polar Electro, Kempele, Finland) and 6–20 Borg scale [22]. Participants recorded HR and RPE data during the unsupervised sessions in a training log. The MICT group trained continuously for 30 min at 60–70% peak heart rate (HRpeak)/RPE of 11–13 on the Borg Scale, whereas each 4HIIT and 1HIIT session began with a 10-min warm-up and concluded with a 3-min cool-down at 50–70% HRpeak. The 4HIIT intervention included four bouts of 4-min intervals performed at 85–95% HRpeak/RPE of 15–17 on the Borg scale, interspersed with 3-min of active recovery performed at 50–70% HRpeak, totaling 38 min per session. The 1HIIT intervention comprised of one 4-min bout of exercise performed at 85–95% HRpeak/RPE of 15–17 on the. Borg scale, totaling 17-min per session.

### Statistical Analysis

Data were analysed using the SPSS version 25 package (IBM, New York, NY, USA). Chi-square tests were used to compare exercise adherence between exercise intervention groups. Analysis of covariance (ANCOVA) was used to determine the between-group difference in the change in continuous variables from pre- to post-intervention, with the change-value assigned as the dependent variables and the baseline value as the covariate. Eta squared (*η*^2^) group x time interaction effect sizes were calculated as between-group sum of squares divided by the total sum of squares and interpreted as follows: ‘small’ effect (0.01); ‘small-to-medium’ effect (0.01 to 0.10); ‘medium-to-large’ effect (0.10 to 0.25) [23]. Continuous variables are presented as mean ± standard deviation or median (range), whilst categorical variables are reported as frequencies.

To determine individual FFI training responsiveness, delta values (post-intervention value minus pre-intervention value) were calculated. A participant was considered a likely responder if the delta FFI value was ≥ 1 unit. Chi square tests were used to analyse the proportion of training response for FFI with subsequent Cramer’s V test to quantify effect size. Significance level was set at *p* < 0.05.

## Results

Seventy-seven out of the 99 participants recruited as part to the EX-MET trial conducted from January 2013 to August 2015 had complete pre- and post-intervention data to determine the primary outcome of the study (Fig. 1). Table 1 provides the baseline data of the 77 participants. The MICT, 4HIIT, and 1HIIT groups completed 89 ± 13%, 88 ± 10%, and 89 ± 14% of the prescribed training sessions, respectively (group difference, *p* = 0.54). There were no reported physical injuries that were directly related to the prescribed exercise interventions.

**Table 1:** Participants’ characteristics

Variable	MICT (n = 26)	4HIIT (n = 25)	1HIIT (n = 26)
**Demographics**			
Age, years (mean ± SD)	55.0 ± 9.8	57.1 ± 9.2	57.1 ± 7.4
Male, sex (%)	69	52	65
Type 2 diabetes (%)	35	44	46
Hypertensive (%)	73	76	77
Weight, kg [mean ± SD or median (range)]	98.2 ± 16.8	91.5 (13.5)	92.4 ± 20.1
MVPA time	57.5 ± 24.3	56.6 ± 20.2	53.3 ± 26.2
**Medications**			
ACEIs, %	46	48	50
Calcium antagonist, %	8	32	8
Beta-blocker, %	12	4	15
Statin, %	40	56	54
Acetylsalicylic, %	19	28	23
Metformin, %	31	32	35

*MVPA* moderate to vigorous physical activity; *MICT* moderate-intensity continuous training; *4HIIT* 4 × 4 min high-intensity interval training; *1HIIT* 1 × 4 min high-intensity interval training; *ACEIs* angiotensin-converting enzyme inhibitors; *SD* standard deviation

### Fitness-Fatness Index

Table 2 presents FFI changes from pre- to post exercise training. While there was no significant between group difference (*p* = 0.30), there was a small group x time interaction effect on FFI [*F*(2, 73) = 1.226; *p* = 0.30; *η*^2^ = 0.01] with the magnitude of FFI increase from baseline shown to be numerically higher in the HIIT groups (4HIIT, + 16%; 1HIIT, + 11%) compared to MICT (+ 7%). A similar trend was found when comparing those without T2D (4HIIT, + 15%; 1HIIT, + 11%; MICT, + 7%; between groups, *p* = 0.83; Table 3) or with T2D (4HIIT, + 17%; 1HIIT, + 10%; MICT, + 5%; *p* = 0.21; Table 4).

**Table 2:** All participants—changes in Fitness-Fatness Index, metabolic syndrome risk factors, and body composition following the exercise interventions

Outcome variables	MICT (n = 26)		4HIIT (n = 25)		1HIIT (n = 26)			
	Baseline	Post	Baseline	Post	Baseline	Post	Between group difference (*p*-value)	Between group difference (Effect size; *η*^2^)
**Fitness-Fatness Index**								
FFI (METs/WtHR)	13.3 ± 4.7	14.2 ± 5.0	11.8 ± 2.9	13.7 ± 3.8	12.8 ± 3.5	14.2 ± 3.6	0.30	0.01
METs	7.9 ± 2.3	8.3 ± 2.3	7.0 ± 1.5	8.0 ± 1.9	7.6 ± 1.8	8.2 ± 1.9	0.26	0.01
Relative V̇O_2_peak (mL/kg/min)	27.6 ± 7.9	28.9 ± 8.0	24.6 ± 5.3	28.1 ± 6.8	26.5 ± 6.3	28.8 ± 6.7	0.25	0.01
Absolute V̇O_2_peak (L/min)	2.7 ± 0.8	2.8 ± 0.8	2.3 ± 0.6	2.6 ± 0.8	2.4 ± 0.7	2.6 ± 0.8	0.30	0.01
WtHR	0.61 ± 0.1	0.60 ± 0.1	0.60 ± 0.1	0.59 ± 0.1	0.60 ± 0.1	0.59 ± 0.1	0.82	< 0.001
**Metabolic Syndrome Risk Factors**								
Triglycerides (mmol/L)	1.6 (0.7 to 6.6)	1.6 (0.7 to 5.2)	1.8 (0.6 to 6.5)	1.8 (0.7 to 4.6)	2.0 (0.7 to 2.8)	1.6 (0.6 to 3.0)	0.86	0.002
HDL-C (mmol/L)	1.1 ± 0.4	1.2 ± 0.4	1.0 (0.4)	1.2 ± 0.4	1.2 ± 0.4	1.3 ± 0.4	0.50	0.01
LDL-C (mmol/L)	2.9 ± 0.8	2.7 ± 0.9	2.3 (0.2 to 6.5)	2.4 (1.3 to 6.7)	2.4 (1.1 to 6.5)	2.3 (1.0 to 4.7)	0.22	0.02
Total cholesterol (mmol/L)	4.8 ± 1.0	4.6 ± 1.0	4.1 (2.6 to 9.3)	4.6 (2.9 to 9.1)	4.5 (3.1 to 9.3)	4.2 (2.9 to 6.9)	0.06	0.03
Waist circumference (cm)	107 ± 12	105 ± 12	104 ± 10	102 ± 9	103 ± 12	101 ± 12	0.64	0.002
Systolic BP (mm Hg)	132 ± 12	126 ± 11	128 ± 14	129 ± 11	136 ± 16	128 ± 15	0.16	0.03
Diastolic BP (mm Hg)	87 ± 9	82 ± 8	83 ± 8	80 ± 7	82 ± 10	79 ± 10	0.88	0.003
Fasting glucose (mmol/L)	5.8 (4.6 to 16.4)	5.7 (4.1 to 12.4)	6.2 (3.6 to 13.6)	5.6 (4.4 to 12.7)	6.2 (4.3 to 13.0)	6.0 (4.4 to 14.3)	0.50	0.01
**Body composition**								
Weight (kg)	98.2 ± 16.8	97.4 ± 17.7	91.5 (13.5)	90.0 (13.5)	92.4 ± 20.1	91.0 ± 19.4	0.63	0.001
Hip Circumference (cm)	114 ± 12	114 ± 13	113 ± 12	113 ± 11	110 ± 11	110 ± 12	0.83	0.001
Total Body Fat (%)	38.7 ± 9.0	38.2 ± 9.1	40.8 ± 7.4	40.2 ± 7.6	39.1 ± 6.9	38.6 ± 7.0	0.97	< 0.001
Trunk Fat (%)	42.1 ± 8.5	41.5 ± 8.4	43.7 ± 6.7	43.1 ± 7.0	42.6 ± 6.1	42.1 ± 5.9	0.97	< 0.001
Android Fat (%)	44.4 ± 8.0	44.0 ± 7.8	45.8 ± 6.5	45.1 ± 6.8	44.9 ± 5.1	44.2 ± 5.3	0.93	< 0.001
Gynoid Fat (%)	37.5 ± 9.7	37.3 ± 9.9	40.0 ± 7.8	39.2 ± 8.4	37.5 ± 7.9	36.9 ± 8.2	0.34	0.001
Lean Body Mass (kg)	56.9 ± 11.3	56.7 ± 11.4	52.5 ± 9.8	52.3 ± 9.8	53.4 ± 12.2	53.2 ± 12.5	0.66	< 0.001
BMI (kg/m^2^)	33 ± 6	32 ± 5	32 ± 6	33 ± 5	32 ± 5	32 ± 5	0.84	0.004

*MICT* moderate-intensity continuous training; *4HIIT* 4 × 4 min high-intensity interval training; *1HIIT* 1 × 4 min high-intensity interval training; *FFI* Fitness-Fatness Index; *MET* metabolic equivalent; *V̇O*_*2*_*peak* peak oxygen uptake; *WtHR* waist circumference-to-height ratio; *HDL-C* high-density lipoprotein-cholesterol; *LDL-C* low-density lipoprotein-cholesterol; *BP* blood pressure; *BMI* body mass index; *SD* standard deviation

**Table 3:** Non-T2D participants—changes in Fitness-Fatness Index, metabolic syndrome risk factors, and body composition following the exercise interventions

Outcome variables	MICT (n = 17)		4HIIT (n = 14)		1HIIT (n = 14)			
	Baseline	Post	Baseline	Post	Baseline	Post	Between group difference (*p*-value)	Between group difference (Effect size; *η*^2^)
**Fitness-Fatness Index**								
FFI (METs/WtHR)	14.1 ± 4.7	15.1 ± 5.3	11.7 ± 2.2	13.5 ± 2.9	12.3 ± 3.4	13.7 ± 3.5	0.83	0.003
METs	8.3 ± 2.2	8.7 ± 2.4	7.0 ± 1.2	7.9 ± 1.8	7.3 ± 1.8	8.0 ± 2.0	0.74	0.01
Relative V̇O_2_peak (mL/kg/min)	29.0 ± 7.8	30.5 ± 8.3	24.4 ± 4.1	27.8 ± 6.2	25.7 ± 6.1	27.9 ± 6.9	0.72	0.01
Absolute V̇O_2_peak (L/min)	2.8 ± 0.8	2.9 ± 0.8	2.3 ± 0.6	2.5 ± 0.9	2.4 ± 0.8	2.6 ± 0.9	0.61	0.005
WtHR	0.60 ± 0.07	0.59 ± 0.08	0.60 ± 0.05	0.59 ± 0.04	0.60 ± 0.1	0.59 ± 0.05	0.83	< 0.001
**Metabolic syndrome risk factors**								
Triglycerides (mmol/L)	1.6 (0.7 to 4.0)	1.5 (0.65 to 4.91)	1.9 (1.1 to 6.5)	1.7 (0.7 to 4.2)	1.7 ± 0.7	1.6 ± 0.6	0.68	0.01
HDL-C (mmol/L)	1.2 ± 0.4	1.2 ± 0.3	1.2 ± 0.4	1.2 ± 0.4	1.4 ± 0.4	1.4 ± 0.4	0.63	0.01
LDL (mmol/L)	3.0 ± 0.8	2.9 ± 0.9	2.6 ± 1.1	2.9 ± 1.0	3.3 ± 1.4	3.0 ± 1.2	0.34	0.03
Total cholesterol (mmol/L)	5.0 ± 1.0	4.8 ± 1.0	4.7 ± 1.1	5.0 ± 1.2	4.9 (3.8 to 9.3)	5.1 (3.6 to 6.9)	0.36	0.02
Waist circumference (cm)	106 ± 13	104 ± 14	102 ± 11	100 ± 7	104 ± 14	100 ± 13	0.64	0.004
Systolic BP (mm Hg)	131 ± 14	125 ± 11	133 ± 16	130 ± 12	132 ± 15	123 ± 9	0.16	0.07
Diastolic BP (mm Hg)	87 ± 11	83 ± 8	85 ± 9	82 ± 8	82 ± 13	79 ± 11	0.82	0.01
Fasting glucose (mmol/L)	5.6 ± 0.7	5.5 ± 0.6	5.5 ± 0.9	5.4 ± 0.6	5.7 ± 0.8	5.5 ± 0.7	0.82	0.01
**Body composition**								
Weight (kg)	97 ± 20	97 ± 21	91 ± 17	89 ± 13	94 ± 21	92.0 ± 20	0.43	0.002
Hip Circumference (cm)	115 ± 13	115 ± 14	113 ± 8	112 ± 9	113 ± 10	112 ± 10	0.99	< 0.001
Total Body Fat (%)	39.6 ± 9.5	39.5 ± 9.7	42.1 ± 6.3	41.7 ± 5.7	41.8 ± 6.1	41.1 ± 6.2	0.67	0.001
Trunk Fat (%)	42.9 ± 8.9	42.7 ± 8.9	44.5 ± 5.6	44.2 ± 4.6	44.6 ± 5.2	43.9 ± 5.0	0.87	0.001
Android Fat (%)	45.4 ± 8.5	45.5 ± 8.2	46.8 ± 4.2	46.7 ± 3.6	46.9 ± 4.0	46.2 ± 4.1	0.40	0.005
Gynoid Fat (%)	39.0 ± 10.2	38.9 ± 10.6	41.5 ± 7.2	41.1 ± 7.2	41.1 ± 7.2	40.5 ± 7.4	0.56	0.001
Lean Body Mass (kg)	55.4 ± 12.3	55.1 ± 12.7	50.2 ± 10.5	49.3 ± 9.6	52.2 ± 14.3	52.2 ± 14.6	0.26	0.001
BMI (kg/m^2^)	32 ± 6	32 ± 6	31 ± 5	33 ± 5	33 ± 5	33 ± 4	0.82	0.01

*MICT* moderate-intensity continuous training; *4HIIT* 4 × 4 min high-intensity interval training; *1HIIT* 1 × 4 min high-intensity interval training; *FFI* Fitness-Fatness Index; *MET* metabolic equivalent; *V̇O*_*2*_*peak* peak oxygen uptake; *WtHR* waist circumference-to-height ratio; *HDL-C* high-density lipoprotein-cholesterol; *LDL-C* low-density lipoprotein-cholesterol; *BP* blood pressure; *BMI* body mass index; *SD* standard deviation

**Table 4:** T2D participants—changes in Fitness-Fatness Index, metabolic syndrome risk factors, and body composition following the exercise interventions

Outcome variables	MICT (n = 9)		4HIIT (n = 11)		1HIIT (n = 12)			
	Baseline	Post	Baseline	Post	Baseline	Post	Between group difference (*p*-value)	Between group difference (Effect size; *η*^2^)
**Fitness-Fatness Index**								
FFI (METs/WtHR)	11.7 ± 4.6	12.3 ± 4.1	11.9 ± 3.7	13.9 ± 4.9	13.4 ± 3.8	14.7 ± 3.9	0.21	0.02
METs	7.1 ± 2.2	7.4 ± 1.9	7.1 ± 1.9	8.1 ± 2.2	7.9 ± 1.9	8.5 ± 1.9	0.19	0.02
Relative V̇O_2_peak (mL/kg/min)	25.0 ± 7.8	26.0 ± 6.8	24.7 ± 6.8	28.4 ± 7.7	27.5 ± 6.5	29.8 ± 6.5	0.19	0.02
Absolute V̇O_2_peak (L/min)	2.4 ± 0.6	2.5 ± 0.5	2.4 ± 0.6	2.7 ± 0.7	2.4 ± 0.6	2.6 ± 0.6	0.37	0.02
WtHR	0.63 ± 0.1	0.62 ± 0.1	0.61 ± 0.1	0.60 ± 0.1	0.60 ± 0.1	0.59 ± 0.1	0.75	< 0.001
**Metabolic syndrome risk factors**								
Triglycerides (mmol/L)	1.6 (1.1 to 6.6)	1.6 (1.0 to 5.2)	1.7 (0.6 to 3.5)	1.8 (1.0 to 4.6)	1.8 ± 0.8	1.6 ± 0.6	0.24	0.03
HDL-C (mmol/L)	1.0 ± 0.3	1.1 ± 0.6	1.0 (0.6 to 2.1)	0.9 (0.9 to 2.2)	1.0 ± 0.3	1.1 ± 0.2	0.64	0.02
LDL (mmol/L)	2.7 ± 0.9	2.4 ± 0.7	1.7 (0.2 to 6.5)	1.8 (1.3 to 6.7)	2.1 ± 1.0	2.0 ± 0.7	0.65	0.01
Total cholesterol (mmol/L)	4.5 ± 1.0	4.2 ± 1.0	3.5 (2.6 to 9.3)	3.8 (2.9 to 9.1)	3.4 (3.1 to 7.2)	3.8 (2.9 to 5.5)	0.17	0.04
Waist circumference (cm)	109 ± 8	107 ± 8	105 ± 10	104 ± 10	103 ± 11	101 ± 11	0.90	0.001
Systolic BP (mm Hg)	134 ± 8	127 ± 10	123 ± 7	128 ± 9	141 ± 17	134 ± 18	0.03	0.10
Diastolic BP (mm Hg)	87 ± 6	82 ± 7	81 ± 6	78 ± 7	81 ± 6	79 ± 8	0.96	0.003
Fasting glucose (mmol/L)	6.7 (5.6 to 16.4)	6.6 (4.3 to 12.4)	6.6 (5.1 to 13.6)	7.4 (5.2 to 12.7)	7.7 (4.3 to 2.9)	6.6 (6.0 to 14.3)	0.41	0.03
**Body composition**								
Weight (kg)	100 ± 9	99 ± 11	94 (82 to 138)	92 (83 to 135)	91 ± 20	90 ± 20	0.99	< 0.001
Hip Circumference (cm)	111 ± 10	112 ± 11	114 ± 15	113 ± 14	107 ± 12	108 ± 15	0.61	0.01
Total Body Fat (%)	36.9 ± 8.3	35.8 ± 7.7	39.3 ± 8.6	38.3 ± 9.5	35.7 ± 6.6	35.4 ± 6.9	0.48	0.003
Trunk Fat (%)	40.7 ± 7.9	39.1 ± 7.1	42.7 ± 8.1	41.6 ± 9.2	40.0 ± 6.3	39.7 ± 6.3	0.54	0.004
Android Fat (%)	42.5 ± 7.1	41.2 ± 6.6	44.4 ± 8.7	43.1 ± 9.4	42.3 ± 5.4	41.8 ± 5.7	0.51	0.01
Gynoid Fat (%)	34.8 ± 8.3	34.4 ± 8.3	38.1 ± 8.6	36.7 ± 9.6	33.0 ± 6.4	32.5 ± 7.2	0.33	0.004
Lean Body Mass (%)	59.8 ± 9.1	59.8 ± 8.2	55.5 ± 8.3	56.1 ± 9.0	54.9 ± 9.4	54.5 ± 9.8	0.31	0.002
BMI (kg/m^2^)	34(26 to 44)	34 (24 to 36)	30 (23 to 43)	30.0 (28 to 42)	31 ± 6	31 ± 5	0.91	0.005

*MICT* moderate-intensity continuous training; *4HIIT* 4 × 4 min high-intensity interval training; *1HIIT* 1 × 4 min high-intensity interval training; *FFI* Fitness-Fatness Index; *MET* metabolic equivalent; *V̇O*_*2*_*peak* peak oxygen uptake; *WtHR* waist circumference-to-height ratio; *HDL-C* high-density lipoprotein-cholesterol; *LDL-C* low-density lipoprotein-cholesterol; *BP* blood pressure; *BMI* body mass index; *SD* standard deviation

**Fig. 2:**
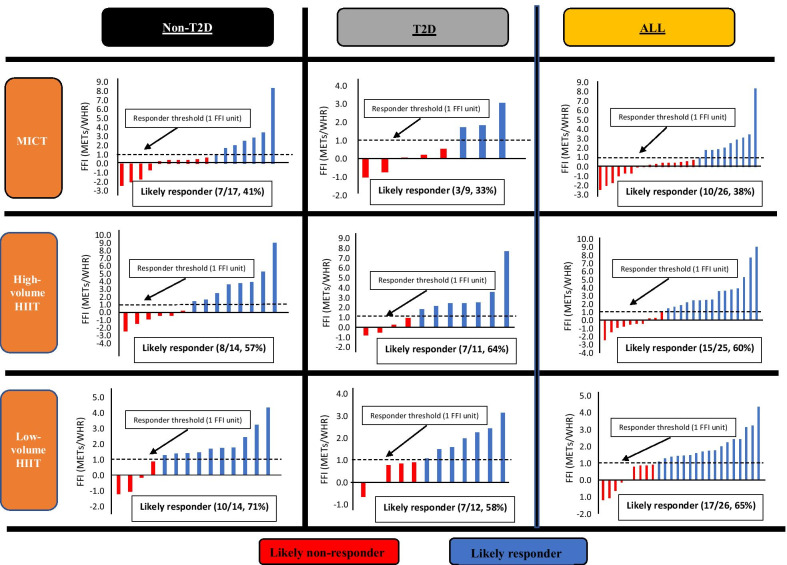
Proportions of response categories in FFI change following exercise interventions in participants diagnosed with MetS with or without T2D

Figure 2 presents the proportion of likely responders and likely non-responders to a clinically meaningful change in FFI. In all participants, there was a numerically greater proportion of participants who responded to a clinically meaningful change in FFI following high-volume HIIT (60%, 15/25) and low-volume HIIT (65%, 17/26) compared to MICT (38%, 10/26), but with no significant between-group difference (*p* = 0.12). A sub-analysis that compared participants with or without T2D showed that low-volume HIIT (71%, 10/14) induced a greater proportion of likely responders to a ‘clinical change’ in FFI (1 FFI unit increase) compared to MICT (41%, 7/17) and high-volume HIIT (57%, 8/14) in those without T2D, but with no significant between-group difference (*p* = 0.24), whereas in those with T2D, MICT (33%, 3/9) had a numerically lower proportion of likely responders to a clinically significant change in FFI compared to high-volume HIIT (64%, 7/11) and low-volume HIIT (58%, 7/12), with no between-group difference (*p* = 0.36).

Tables 2, 3, 4 show a similar pattern of change in relative V̇O_2_peak within- and between-groups, with small changes in WtHR following the exercise programs.

### Body Composition and Fasting Lipid Profile

Tables 2, 3, 4 present negligible changes in total body fat (MICT, − 1%; 4HIIT, − 1%; 1HIIT, − 1%), trunk fat (MICT, − 1%; 4HIIT, − 1%; 1HIIT, − 1%), android fat (MICT, − 1%; 4HIIT, − 2%; 1HIIT, − 2%), and gynoid fat (MICT, − 1%; 4HIIT, − 2%; 1HIIT, − 2%) following all exercise interventions. Tables 2, 3, 4 also show negligible changes in blood lipid profile following all exercise interventions. There were no significant between- and within-group changes in total energy intake from baseline to post-intervention.

### Moderate-to-Vigorous Physical Activity

There was a significant MVPA change between training groups following the intervention period (MICT, + 66%; 4HIIT, + 12%; 1HIIT, − 2%, *F*(2.42) = 4.89, *p* = 0.01). Post hoc analysis revealed that MICT significantly increased MVPA more than 1HIIT (*p* = 0.02). Accelerometer wear time from pre- to post-intervention (*p* = 0.085) had no significant change (*p* = 0.85).

## Discussion

This is the first study to investigate changes in FFI following different exercise volumes in adults with MetS. There were no statistically significant difference in FFI changes between exercise groups. However, it should be highlighted that HIIT, regardless of the training volume (high-volume HIIT, 114 min/week; low-volume HIIT, 51 min/week) induced a greater numerical proportion of likely responders to a ‘clinically’ significant improvement in FFI (1 FFI unit increase) (high volume HIIT; 60%; low volume HIIT, 65%) compared to 150 min per week of MICT (38%). This is an important finding as it has been reported that only about 30% of the Australian population participate in regular exercise (Brown et al. 2002), with time deficiency as the most reported culprit (Trost et al. 2002).

Consistent with a previous study [9], the proportion of participants who met the clinical threshold to a meaningful FFI change in the present study appears to be driven by an increase in fitness, rather than a reduction in fatness. Our study also showed a similar pattern in inter-individual V̇O_2_peak changes between exercise groups, whereas WtHR showed negligible change magnitude from pre-to post-intervention. This is further supported by the lack of significant changes in our body fat indices derived via a DEXA scan which is regarded as a robust method of assessing body composition [24]. Williams et al. [25] also found a similar trend in inter-individual V̇O_2_peak changes relative to the present study, with high-volume HIIT (31%) and low-volume HIIT (16%) also showing more likely responders to a clinically significant improvement in V̇O_2_peak compared to MICT (21%).

However, inconsistent with our findings, previous studies have reported favourable changes in body composition and lipid profile following similar exercise regimens [26, 27]. The lack of body composition and lipid profile improvements from pre- to post-intervention in the present study could be attributed to the absence of total energy intake reduction following the exercise programs. This notion is supported by investigations which have shown superior capacity for combined exercise and diet intervention compared to exercise alone in improving body composition [28, 29] and lipid profile in overweight to obese adults [29]. Nevertheless, there is evidence to suggest that short-term HIIT (8–12-weeks) without caloric restriction is sufficient to induce favourable changes in body composition and lipid profile in young (18–21 years old) previously sedentary overweight or obese males [27, 30]. Further investigations on the impact of HIIT and MICT on body composition and lipid profile, specifically in adults with metabolic syndrome, are therefore warranted.

In parallel with a clinical FFI change, the present study also found a greater number of participants in the HIIT groups who reversed the MetS (*n* = 9) compared to the MICT group (*n* = 1), which was also previously reported by our group [31]. Although MetS significantly increases one’s risk of CVD-related mortality, it has been reported that fit individuals with MetS are less susceptible to CVD compared to less fit counterparts, despite the existence of central obesity as a component of this syndrome [3]. These findings, therefore, collectively underscore the importance of targeting fitness over fatness in improving cardiovascular health. We hypothesise that the importance of targeting fitness improvement over fat-loss in reducing MetS incidence could be attributable to increased protection against a mismatch between oxygen demand and supply that typically occurs in excess adipose tissue, resulting in hypoxia-induced necrosis of this excess adipose tissue [32]. This could have in turn led to the prevention of subsequent insulin resistance, inflammation, and oxidative stress, which are all factors known to exacerbate and promote the clustering of CVD risk factors constituting the MetS [33].

Our sub-analysis also showed that in those with T2D, there is a similar pattern in inter-individual response to a clinical meaningful FFI change following the different exercise interventions (*n* = 32; MICT, 33%; high-volume HIIT, 64%; low-volume HIIT, 58%). However, in those without T2D (*n* = 45), low-volume HIIT (71%) appeared to induce a greater proportion of likely responders compared to larger exercise volumes (MICT, 41%; 4HIIT, 57%), but with no significant difference between groups. This highlights the potential importance of exercise intensity over exercise duration as a prophalactic against incident T2D and CVD. As little as 4 min of high-intensity exercise performed three times a week should therefore be at least recommended as a preventative strategy to reduce risk of T2D and CVD-related mortality at the population level. Our results are consistent with a previous study [34] which showed that exercise intensity is a more important factor relative to exercise volume in optimizing physiological stress to maximize adaptations of factors contributing to a positive fitness response to training. Our results are also supported by Ross et al. [35], who reported that at fixed amount of exercise (energy expenditure, kcal), increasing exercise intensity results in elimination of non-responders to exercise [35].

It should be noted that we also found a wide variability in FFI changes in response to our 16-week training interventions (MICT, 4HIIT, 1HIIT, Fig. 2). This is in agreement with previous findings that not all individuals, irrespective of baseline status (i.e. age, sex, fat mass, fat free mass, weight, and race) [36, 37], respond positively to a specific dose of standardized exercise, with considerable individual variability in training adaptations including so-termed ‘non-responders’ and, in some cases, ‘adverse responders’. The absence of a personalized approach to the exercise prescription has been put forth to explain the variability in response to exercise [38]. It has been purported that a more individualized approach to exercise prescription may enhance training efficacy and limit training unresponsiveness. This notion is supported by Wolpern et al. [39] which showed that when exercise intensity is adjusted according to a ‘personalized prescription’ or threshold-based model (i.e. ventilatory threshold), a more favourable change in fitness was evident in 100% of participants compared to only 41.7% when the exercise intensity was ‘standardized’ or prescribed according to a relative per cent method (i.e. % heart rate reserve [HRR]). Indeed, it has been put forth that the response variability following a ‘standardized exercise prescription’ may be attributable to the inability of this method to account for individual metabolic difference [40]. It is plausible that the standardized exercise dose implemented in the present study and others [40] is insufficient to overcome the threshold to promote fitness improvement or exercise responders in all participants. Likewise, a standardized exercise prescription-induced ‘adverse response’ may also result from an overestimation or underestimation of the required exercise dosage to foster a positive outcome.

### Limitations

The main limitation of this study is the sample size, which could explain the lack of statistically significant differences found between groups. The lack of a control group to determine within subject variation should also be considered as a major limitation of this study, limiting our ability to determine if the identified change is beyond the variability and technical measurement error of the desired outcome measure. Our results should therefore be taken with caution until larger clinical trials are conducted. Another study limitation worth mentioning is the standardized protocol (% heart rate peak and RPE) used to prescribe the intensity of the exercise interventions, possibly influencing the variability noted in the exercise response. As previously mentioned, it would have been more informative to personalize the intensity prescription using a threshold-based model, for example. Future studies are encouraged to utilize this prescription method to determine its impact on the exercise response.

## Conclusion

The main finding was that exercise intensity may affect the responsiveness of individuals to improvements in FFI, when numerical differences between exercise groups are considered. Specifically, our study shows that HIIT, regardless of the training volume may generate a greater numerical proportion of likely responders to clinically significant improvements in FFI compared to MICT. However, it should be noted that there was no statistically significant difference in inter-individual FFI response between exercise interventions.

## Data Availability

The datasets generated and/or analysed during the current study are not publicly available but are available from the corresponding author on reasonable request.

## Abbreviations

MetS: Metabolic syndrome
CVD: Cardiovascular disease
FFI: Fitness-fatness index
EX-MET: Exercise in the prevention of metabolic syndrome
MICT: Moderate-intensity continuous training
4HIIT: High-volume high-intensity interval training
1HIIT: High-volume high-intensity interval training
V̇O_2_peak: Peak oxygen uptake
V̇O_2_: Oxygen uptake
WtHR: Ratio of waist circumference-to-height
METs: Metabolic equivalents
T2D: Type 2 diabetes
DEXA: Dual-energy X-ray absorptiometry
HRpeak: Peak heart rate
RPE: Rate of perceived exertion
ANCOVA: Analysis of covariance
HRR: Heart rate reserve
